# Adapting interventions to new contexts—the ADAPT guidance

**DOI:** 10.1136/bmj.n1679

**Published:** 2021-08-03

**Authors:** Graham Moore, Mhairi Campbell, Lauren Copeland, Peter Craig, Ani Movsisyan, Pat Hoddinott, Hannah Littlecott, Alicia O’Cathain, Lisa Pfadenhauer, Eva Rehfuess, Jeremy Segrott, Penelope Hawe, Frank Kee, Danielle Couturiaux, Britt Hallingberg, Rhiannon Evans

**Affiliations:** 1 Centre for Development, Evaluation, Complexity and Implementation in Public Health improvement (DECIPHer), School of Social Sciences, Cardiff University, Cardiff, UK; 2 MRC/CSO Social and Public Health Sciences Unit, University of Glasgow, Glasgow, UK; 3 Pettenkofer School of Public Health, Munich, Germany; 4 Institute for Medical Information Processing, Biometry and Epidemiology, LMU Munich, Munich, Germany; 5 Nursing, Midwifery and Allied Health Professions Research Unit, University of Stirling, Stirling, UK; 6 School for Health and Related Research, University of Sheffield, Sheffield, UK; 7 Centre for Development, Evaluation, Complexity and Implementation in Public Health improvement (DECIPHer), Centre for Trials Research, Cardiff University, Cardiff, UK; 8 Menzies Centre for Health Policy, Sydney School of Public Health, University of Sydney, Sydney, NSW, Australia; 9 O’Brien Institute of Public Health, University of Calgary, Calgary, AB, Canada; 10 Centre for Public Health, Queens University Belfast, Belfast, UK; 11 Cardiff School of Sport and Health Sciences, Cardiff Metropolitan University, Cardiff, UK

## Abstract

Implementing interventions with a previous evidence base in new contexts might be more efficient than developing new interventions for each context. Although some interventions transfer well, effectiveness and implementation often depend on the context. Achieving a good fit between intervention and context then requires careful and systematic adaptation. This paper presents new evidence and consensus informed guidance for adapting and transferring interventions to new contexts.

Interest is growing in adapting evidence informed interventions for implementation in new contexts.^1^ ^2^ ^3^ ^4^ Box 1 provides a list of definitions of key terms. Use of interventions with an existing evidence base could be more efficient than the development of new interventions for each context. However, effects often depend on the context.^6^ Hence, interventions that are simply replicated might be less likely to reproduce effects than those adapted to achieve a good fit between intervention and context.^7^ Situations in which adaptation might be needed include transporting an intervention to a new setting^8^ or targeting different populations, such as adapting for sociocultural groups^9^ (box 2 lists examples). Adaptations might aim to avoid inequalities generated from interventions, by ensuring that interventions delivered at the population level are sensitive to the needs of disadvantaged groups.^10^ When effects are not reproduced in new contexts, however, it can be difficult to determine whether this result is due to inappropriate adaptation, weaknesses in original evidence, mechanisms that do not function in the new context, or another explanation.

Box 1 **Definitions of key terms**

**Adaptation**

Intentional modification(s) of an evidence informed intervention, in order to achieve a better fit between an intervention and a new context. Modification can include planned adaptations (changes made before introducing a new intervention) and responsive adaptations (changes made intentionally, but in response to emerging contextual issues occurring during implementation). Adaptation of interventions is likely to be ongoing as context changes over time.

**Evidence informed intervention**

Interventions that already have existing evidence from another context. Although the term “evidence based interventions” tends to emphasise effectiveness, we consider evidence informed interventions to include interventions with previous evidence showing that the intervention has worked in changing outcomes of interest (or different outcomes); or with evidence relating to feasibility, acceptability, delivery processes, or cost effectiveness.

**Implementation**

The delivery of evidence informed interventions in routine practice. Implementation considerations run through all stages of intervention research; for example, a process evaluation studying delivery in order to understand how implementation in routine practice might be achieved or undermined.

**Context**

Any feature of the circumstances in which an intervention is implemented that might interact with the intervention to produce variation in outcomes. Important aspects of context might include, but are not limited to, geographical, organisational or service, cultural, economic, ethical, legal, and political circumstances, and local practices.^5^ These features of context change to some extent over time, as well as between locations.

Box 2 **Examples of types of intervention adaptation**

**Adapting interventions for a new setting or healthcare system**

Divan and colleagues used an intervention for children with autism that had originally been developed and evaluated in higher income contexts and was delivered by speech and language therapists.^8^ As well as cultural adaptation, transferring this intervention to South Asian contexts involved an emphasis on task shifting, integrating the intervention into the roles of non-specialists.

**Adapting interventions to improve impacts for population subgroups**

Burrow-Sanchez and colleagues adapted a group intervention based on cognitive behavioural therapy for substance abuse treatment for Latino adolescents.^9^ Overall effects were similar to the original intervention, although the adapted intervention had stronger effects for young people who had greater commitment to a Latino ethnic identity.

Efforts to examine what kinds of adaptation enhance the likelihood of interventions working in new contexts have proven inconclusive owing to limited transparency in conduct and reporting of adaptation.^7^ ^11^ In the ADAPT study,^12^ funded by the UK Medical Research Council and National Institute for Health Research, we developed guidance to improve the conduct and reporting of intervention adaptations. Our guidance focuses on involving stakeholders in adaptation, selecting a suitable evidence informed intervention, planning and undertaking adaptations, evaluating adapted interventions, implementing adapted interventions in routine practice, and reporting adaptation processes and outcomes.

**Summary points**

Use of interventions with a previous evidence base in new contexts might be more efficient than developing new interventions
Many population health problems and interventions are highly sensitive to context, so implementing an intervention in a new context without adaptation might be less likely to lead to positive outcomes
A new consensus informed guidance for adapting interventions to achieve a good fit between the intervention and context (ADAPT) proposes systematic processes for adapting interventions to new contexts, and transparent reporting to facilitate synthesis on what does or does not work
The ADAPT guidance was developed using systematic review methods, qualitative interviews, extensive consultation, and formal consensus methods. It provides a framework and step-by-step guidance for working with stakeholders, selecting suitable interventions, undertaking adaptations, making decisions on evaluation and implementation, and reporting adapted interventions

## How was the ADAPT guidance developed?

The ADAPT guidance was developed using a systematic review of existing guidance^13^ and scoping review of adaptation studies^14^; qualitative interviews with researchers, funders, journal editors, and policy or practice stakeholders^15^; and a three round Delphi consensus exercise (protocol paper^16^). Individual substudies were submitted for publication as completed, with draft guidance developed through discussion and synthesis of findings across work packages by the author group. An advisory group of academic, policy, and practitioner stakeholders participated in an expert panel workshop before the Delphi and provided guidance throughout. A full draft of the guidance was shared and discussed at a series of online workshops with a subset of Delphi participants, and refined. This paper summarises our recommendations; detailed guidance and description of its development is available elsewhere.^17^ Box 3 provides details of our systematic review as well as information on additional tools that emerged after the review’s completion but informed the guidance.

Box 3 **ADAPT systematic review and more recent emerging tools and frameworks**

**Systematic review conducted as part of the ADAPT study**

Our systematic review provides an overview of existing guidance for adapting interventions to new contexts, published up to and including 2018:

- Movsisyan A, Arnold L, Evans R, et al. Adapting evidence-informed complex population health interventions for new contexts: a systematic review of guidance. *Implement Sci* 2019;14:105.^13^

**More recent tools that have influenced ADAPT guidance development**

Assessment of the rationale for intervention, and consideration of the fit between the intervention and context:

- Munthe-Kaas H, Nøkleby H, Lewin S, et al. The TRANSFER approach for assessing the transferability of systematic review findings. *BMC Med Res Method* 2020;20:11.^18^
- Duggleby W, Peacock S, Ploeg J, et al. Qualitative research and its importance in adapting interventions. *Qual Health Res* 2020:1049732320920229.^19^

Planning for and undertaking adaptations:

- Kirk MA, Moore JE, Wiltsey-Stirman S, et al. Towards a comprehensive model for understanding adaptations’ impact: the model for adaptation design and impact (MADI). *Implement Sci* 2020;15:56.^2^

Planning for and undertaking evaluations:

- Bonell C, Prost A, Melendez-Torres G, et al. Will it work here? A realist approach to local decisions about implementing interventions evaluated as effective elsewhere. *J Epidemiol Community Health* 2021;75:46-50.^20^
- Miller CJ, Wiltsey‐Stirman S, Baumann AA. Iterative decision‐making for evaluation of adaptations (IDEA): a decision tree for balancing adaptation, fidelity, and intervention impact. *J Community Psychol* 2020.^3^

Reporting adaptations:

- Wiltsey-Stirman S, Baumann AA, Miller CJ. The FRAME: an expanded framework for reporting adaptations and modifications to evidence-based interventions. *Implement Sci* 2019;14:58.^4^

**Full guidance document**

Our guidance document provides an overview and discusses a broad range of frameworks for considering factors such as mapping similarities and differences between contexts (https://decipher.uk.net/portfolio/the-adapt-study/).^17^

## Framework and recommendations

**Fig 1:**
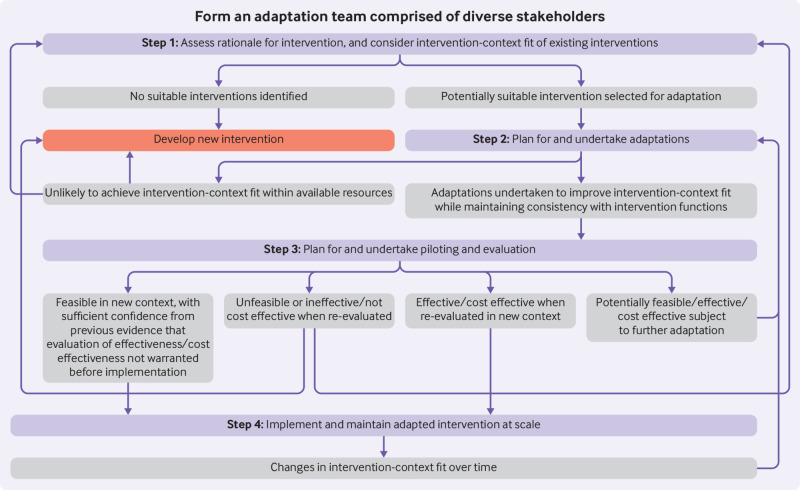
ADAPT process model for adapting interventions to new contexts. Purple boxes=stages of ADAPT step-by-step guidance (box 3 provides more details). Grey boxes=potential outcomes from each stage. Directional arrows=recommendations for moving, forward, or backwards through stages (or exiting)

The ADAPT process model (fig 1), presents the steps of our framework. Outcomes of each step, indicated in the grey boxes, inform decisions on movement forward or backwards, or exiting adaptation processes in favour of developing a new intervention or considering different interventions.

The distinction between intervention development and adaptation is not always clear; development can involve the combination of evidence informed components into a new intervention. Teams might also use a hybrid approach to development and adaptation, in which one intervention is adapted and augmented with new components. In such circumstances, we recommend the use of our guidance in combination with guidance for intervention development.^21^ Most adaptation literature focuses on translating an evidence informed intervention into a new context, which has guided our approach. Some researchers, however, suggest that interventions can and should be adapted to context during development and evaluation.^22^ ^23^ Considering adaptability from the earliest stages of development could help ensure that interventions are more resilient to changes in context. Examples of studies that consider adaptability within intervention development and evaluation are increasing,^24^ aided by advances in complex logic modelling.^25^

We include stakeholder involvement as an overarching principle central to all stages of adaptation, rather than a discrete stage; to operationalise this principle, stakeholder involvement is positioned as surrounding all steps within our model (fig 1).

Box 4 presents a checklist of questions for adaptation teams to consider. These questions do not need to be answered sequentially, and many will be answered by the available evidence. Using these questions to guide discussions within adaptation teams can help identify uncertainties for which further research can inform the adaptation process. Consistent with concerns that much existing guidance for adaptation is overly prescriptive,^26^ we present flexible recommendations relevant to stakeholders who might enter at different stages, and iterate between and within stages. We illustrate recommendations with reference to several empirical examples, a broader selection of which appear in our full guidance document.^15^

Box 4 **Checklist of questions to guide teams in adapting an intervention for a new context**

**Forming an adaptation team**

- Have you involved an appropriate range of stakeholders, including those with expertise in the intervention and its evidence base, and those with knowledge of the new context?
- Is your team clear on roles, including who will make decisions on adaptations, when, and how?
- Will you work with the developers of the intervention? If so, how will you manage any conflicts of interest arising from this?
- How might the membership of your adaptation team need to evolve as adaptation progresses?

**Assessing the rationale for intervention, and considering intervention-context fit**

- What is the problem that an intervention seeks to improve in the target population?
- Is there more than one potential evidence informed intervention? If so, are there reasons one might be more suitable than others, such as the relevance of its programme theory and change mechanisms?
- What is known about the selected intervention(s) in terms of programme theory, process, effectiveness, cost effectiveness and implementation in other contexts?
- How robust are any claims that the intervention(s) has worked elsewhere?
- How similar and different are original and new contexts, in terms of issues likely to affect implementation and effectiveness?
- Are there any intellectual property issues which limit use and adaptation of the intervention(s)?

**Planning for and undertaking adaptations**

- What adaptations can you make to respond to constraints and facilitators, while maintaining consistency with intended intervention functions?
- What adaptations need to be made to intervention materials, such as manuals, to capture changes made to the intervention?
- Might interactions with aspects of the new context lead to any new unintended consequences?
- What costs and resources are needed to deliver the adapted intervention?
- Who will deliver the adapted intervention, and how will you recruit them?

**Planning for and undertaking evaluation**

- Given what is known about the intervention, and the likely transferability of previous evidence, what type and extent of re-evaluation is warranted?
- What will be the value of new information to policymakers, practitioners, and other stakeholders?
- What resources are available for re-evaluation?
- Does initial feasibility testing indicate that any further adaptations are needed?
- How will you capture responsive adaptations and decide whether the intervention remains consistent with intended functions and change mechanisms?
- How will you evaluate effectiveness, cost effectiveness, and process, if this is warranted by uncertainty about whether existing evidence will transfer?

**Implementing and maintaining the intervention at scale**

- What long term partnerships and capacity will be needed for maintenance of the intervention?
- How will you monitor whether the intervention continues to be delivered, and maintains its effectiveness, over time in real world practice?

## Cross-cutting principles: form an adaptation team of diverse stakeholders

### Involve stakeholders early and throughout the adaptation process

We recommend involving diverse stakeholders as early as possible, who will act as a working group (or adaptation team) throughout all stages. When adapting a smoking prevention intervention to a new focus on cannabis prevention, Hawkins and colleagues brought together academics and practitioners to jointly adapt existing and produce new intervention activities.^27^ Adaptation will often involve a core team, including members of the public or patient population, policy and practice stakeholders, and researchers, from considering the suitability of candidate interventions and the potential need for adaptation, to full implementation. Experts on specific issues might be consulted on an ad hoc basis at relevant stages.

### Agree principles for decision making and involvement of members

Agreeing principles for leadership and decision making, and anticipated outcomes for all stakeholders early on could reduce the risk of later disagreements.^28^ Some teams will work in a top-down manner, whereas others will operate more from the bottom-up. For example, decision making might require a consensus or majority vote of all those involved or might be the responsibility of a specified subgroup, with others acting as advisers. Coproduction,^27^ which brings together stakeholders with detailed knowledge of theory and evidence, and stakeholders with detailed contextual knowledge, is likely to be important in achieving fit between an existing intervention and a new context.

### Consider the pros and cons of working with original intervention developers

Developers of the original intervention might have detailed knowledge of its workings. However, intervention developers might also have fixed views on how an intervention should be delivered, which could inhibit an adaptation team’s ability to achieve a good fit between the intervention and context. Power imbalances might be introduced in situations where, for example, developers from higher income contexts are influential in deciding what is best for people in lower income contexts. Developers could have financial and non-financial interests in the wider impact of the intervention. Hence, working with developers can be helpful, but risks need careful management.

### Review and update membership of adaptation team as adaptation progresses

The membership of adaptation teams is likely to be fluid, with roles and membership reviewed throughout. For example, while the participation of people who will implement the intervention is important, it might only be possible to identify those people and seek their involvement once an intervention has been selected.

## Step 1: Assess the rationale for intervention, and consider intervention-context fit

### Define the problem in the target population

Before identifying an intervention, we recommend articulating clearly what the problem is, and why an intervention is needed. This will often include understanding the prevalence of a health problem and its distribution among population subgroups, as well as consideration of causes.

### Identify candidate interventions

In some instances, adaptation teams will begin without a predefined intervention in mind, and seek to systematically identify candidate interventions. In other circumstances, intervention developers could have promoted their intervention in new contexts, or a group might have become aware of a potential intervention by other mechanisms. Nevertheless, even if a team already has an intervention in mind, it should critically reflect on whether the intervention does represent the most suitable choice. For some problems and settings, up-to-date reviews comparing effects of different interventions across various contexts will already be available. If none exists, a new review or evidence map might be needed. Systematic review methods are emerging for explicitly considering transferability of evidence to new contexts.^18^ Such reviews are likely to provide useful sources of information for intervention selection processes. If no suitable interventions are identified, developing a new intervention might be warranted.^21^

### Obtain detailed information on the selected intervention and the contexts in which it has been evaluated

Published information might be enough to enable judgments about which interventions are likely to be suitable.^29^ However, gaps will often need to be filled by obtaining manuals, or through contact with developers. Publications from previous evaluations—including data on feasibility, main effects, subgroup analyses,^30^ and processes—will provide insights to inform judgments on transferability. Failures to replicate intervention effects are often attributed to differences in context, but might be due to shallow theorisation (meaning that only surface features of an intervention are reproduced).^31^ Process evaluations,^32^ where conducted and published, could provide vital information on how the intervention worked, to guide replication of intervention mechanisms. Additional research in contexts where the intervention is in use might sometimes be needed to fully theorise the intervention before adaptation. Devlin and Wight undertook qualitative research on an ongoing Italian drug recovery programme, to more fully theorise and contextualise its mechanisms to inform transfer to Scotland.^33^

### Consider the robustness of effectiveness claims

Claims to effectiveness should be critically examined.^34^ Poor quality evaluations with high risk of bias, or evaluations undertaken by the group who developed the intervention, could provide a less persuasive basis for considering an intervention effective than high quality independent evaluations. For example, while failures to replicate initial successes of the Strengthening Families Programme for Youth (SFP 10-14) in independent evaluations might be due to differences in implementation and context, Gorman argues that these might be because significant results in original studies reflected flexible data analysis and reporting practices.^34^ As well as informing selection of an intervention, the quality of existing evaluations could also inform later decisions on whether another full evaluation of effectiveness is warranted. However, outcomes data are often inconclusive rather than providing evidence of no effect,^35^ while process evaluation data could indicate that fixing problems with delivery or context might lead to greater effects. Hence, interventions that have not previously reached the threshold for a conclusion of effectiveness should not automatically be disregarded.

### Map similarity and difference between original and new contexts

Decisions on the nature and extent of adaptation needed to achieve a good fit between an intervention and context will be largely driven by similarity and difference between original and new contexts. Use of an existing theory or framework (or elements of these) to structure thinking about context can be useful.^36^ Common considerations include availability of resources, feasibility of embedding intervention delivery into existing roles, acceptability (to the target population, people delivering the intervention, and the wider public), willingness and ability of local workforce to adopt the intervention, potential fit with local norms, and existing delivery systems. Characterisation of usual practice is also important because interventions similar to current practice might be easier to implement, but might not make as much difference to outcomes.^37^ Additional qualitative research could be beneficial in deepening understandings of a new context to inform adaptation decisions.^19^

An overemphasis on contextual differences to the neglect of similarities, however, can lead to a perception that substantial adaptation is required where it is not. Some contextual differences will be irrelevant, and an intervention could transfer well to seemingly different contexts while failing to transfer to apparently more similar ones. What matters is the presence of contextual contingencies for successful implementation and for the intervention to function as intended in terms of change mechanisms. Hence, focusing on aspects that might be likely to affect these issues will be more efficient than trying to capture all aspects of context. The ASSIST smoking prevention programme, previously evaluated in schools in England and Wales,^38^ had challenges in Scotland at a time when smoking rates had become much lower.^39^ Work is ongoing to adapt this intervention for Colombia.^40^ Although Colombia might first appear more different to the original context than contemporary Scotland, it could be similar in terms of factors that matter for transferability, such as high smoking rates in schools.

### Consider intellectual property issues

Interventions are sometimes branded as a product, limiting the degree of adaptation allowed. Proprietary interventions could incur license fees, making use prohibitively expensive in lower resource settings. If a holder of intellectual property rights is not included as a collaborator in the adaptation process, legal advice might be needed on whether plans contravene intellectual property rights. If constraints are likely to hamper the fit between an intervention and context, negotiation with developers, or iteration backwards to the selection of a different intervention or development of a new intervention, might be necessary. Gomide and colleagues drew on principles from evidence based interventions to create a new open source, web based intervention to prevent smoking in Portuguese, because existing web based interventions were hosted on proprietary platforms, limiting potential adaptation to Portuguese speaking countries.^41^

## Step 2: Plan and undertake adaptations

### Identify and respond to constraints and facilitators

Considering fit between an intervention and a new context might identify a range of constraints and facilitators to achieving sufficient reach, effectiveness or cost effectiveness, adoption, implementation, or maintenance in the new context.^42^ An adaptation team will need to plan how each constraint might be overcome, and what factors might facilitate successful transfer. Where constraints can be overcome or facilitators used, a team could develop a candidate list of potential adaptations, before reaching initial agreement on which to implement. For example, the Nutrition And Physical Activity Self-Assessment for Child Care^43^ intervention was adapted for the United Kingdom.^44^ Adaptations included changing the labelling of self-assessment forms to “review and reflect” to resolve barriers to implementation that might arise from evoking language associated with government inspectors, while the number of action planning goals was reduced owing to concerns with staff capacity. Careful attention is needed when adapting to context that integrity to intervention mechanisms is retained; the Model for Adaptation Design and Impact (MADI) framework offers one recent tool that considers the likely implications of adaptations on intervention impact.^2^ If constraints are too difficult to resolve with the available resources, intervention development (or selection of a different intervention) could be considered.

### Adapt intervention materials

After considering what changes might be required to achieve a good fit between an intervention and context, new materials could be needed, such as an updated programme theory, manual, protocols, and delivery plan. An overview of planned adaptations and their rationale will allow careful consideration of whether adaptations, as a whole, are consistent with or are likely to undermine intervention mechanisms.^2^ If the adaptation team believes that an intervention has undergone sufficient modifications to have become a new intervention (that is, where intervention mechanisms have been substantially changed), we recommend using our guidance in conjunction with guidance for intervention development.^21^

### Consider potential for unintended consequences

Even if an intervention replicates original benefits, interactions with other features of a new context could cause new and unintended consequences.^45^ Hence, ongoing qualitative and quantitative work throughout piloting, evaluation, and scaling-up might help capture unintended consequences and develop plans to mitigate them.

### Consider costs and resources needed for the adapted intervention

Consideration of resources is likely to form part of earlier assessments of differences and similarities between contexts, and to feed into adaptation decisions. Because an adapted intervention will work with differing local resources, costs could differ substantially between contexts. Once a concrete model of the adapted intervention is available and its likely costs understood, economic modelling could help inform decisions about evaluation and implementation.

### Recruit individuals and groups to deliver the intervention

Once a firm decision has been made to proceed with an adapted intervention, a network of individuals, communities, and organisations might need to be recruited and trained to deliver the intervention. If interventions transported internationally are embedded into workforce roles that do not exist in the same form across contexts, then adaptation might need to focus on task shifting, which identifies whether the intervention can be accommodated into roles of another group while remaining effective. An intervention for children with autism spectrum disorder was adapted for contexts in South Asia where fewer speech and language therapists were available, and intervention delivery was integrated into the roles of non-specialists.^8^

## Step 3: Plan and undertake piloting and evaluation

### Consider the extent and type of evaluation warranted

An adaptation team’s judgments regarding the nature, quality, and transferability of previous evidence will inform decisions on what uncertainties remain, and thus what kinds of additional evidence are needed in the new context.^20^ If uncertainty is high, a new evaluation of effectiveness, cost effectiveness, and process might be necessary. If uncertainty is lower, full implementation might be recommended without further evaluation; in this scenario, evaluation could be built into implementation using natural experiment approaches. 

The team will also need to consider the extent to which evaluation contributes to review level evidence on how an intervention functions in different contexts, or primarily informs decision making in the specific new context. In the second scenario, a less controlled evaluation of effects might be sufficient. Decisions on whether to implement, adapt further, or terminate an adapted intervention will be informed by evidence relating to factors including feasibility, effectiveness, and cost effectiveness. However, gathering new data on all of these issues in the new context will not always be necessary.^46^ The Football Fans in Training intervention supports weight loss in men and has been adapted for different international settings and types of sports club following a successful UK trial.^47^ Adaptations range from very minor to substantial, and re-evaluations range from pragmatic non-randomised studies to full randomised trials.^48^

### Consider the value of new information to policy makers, practitioners, and other stakeholders

Gathering more information through evaluation comes at a cost, so the adaptation team should consider the added value for decision making above and beyond information already available. For example, value of information analysis has been used in healthcare to inform decisions about whether to conduct a full evaluation before implementation, or whether the opportunity cost of delaying implementation will outweigh the value of waiting for an evaluation to be completed.^49^

### Consider resources available for evaluation

In contexts where resources to undertake a full evaluation are not available, efforts might need to target the most important uncertainties that are feasible to resolve within a given budget. Such issues could include, for example, uncontrolled assessments of outcomes, and qualitative exploration of unintended consequences.

### Evaluate feasibility and consider further adaptations based on feedback

Informed by the above considerations, some piloting to test feasibility of the adapted intervention, and of plans for evaluation,^50^ is likely to be useful. If effectiveness is not re-evaluated before implementation, data monitoring structures to be integrated into implementation can be piloted and refined. Piloting could result in the team deciding to proceed, that the intervention is unlikely to be beneficial in the new context, or that further adaptation is needed before the intervention can be taken further. The Australian Healthy Dads Healthy Kids weight management programme for primary school aged children and their fathers^51^ was adapted to increase cultural acceptability to a multi-ethnic UK population.^52^ However, during piloting, meeting recruitment targets and identifying skilled programme facilitators proved challenging. A decision was made not to proceed without further adaptation.

### Document and classify responsive adaptations

Change (both pre-planned and continuous) is a characteristic of many interventions and the contexts in which they are implemented.^53^ Interventions that do not continue to adapt could become redundant as context changes. Systematic processes to monitor and reflect on consistency of adaptations with programme theory will help limit the emergence of spontaneous adaptations which compromise intervention mechanisms.^54^ ^55^ In adapting a behaviour change intervention for patients with impaired mobility, Betts and colleagues convened a team including researchers and interventionists who met monthly during delivery to discuss emerging barriers to participation, and agree responsive adaptations.^56^ Developing an agreed process for capturing and categorising responsive adaptations will enable ongoing consideration of changes likely to support, or undermine, the functioning of the intervention. Documenting responsive adaptations can help guide ongoing delivery within the same context and provide useful information for future adaptations in other contexts.

### Undertake evaluation of effectiveness, cost effectiveness, or process (if warranted)

If the adapted intervention is feasible, but substantial uncertainty remains about whether it will be effective in the new context, a full evaluation of effectiveness and cost effectiveness should be considered. A new evaluation could be a randomised controlled trial or other robust outcomes evaluation design, depending on the nature of intervention. Process evaluations^32^ might usefully focus on understanding perceived impacts of adaptations, as well as documenting unintended harms, and understanding mechanisms. This added information could build on aspects of process evaluations conducted in the previous contexts.

## Step 4: Implement and maintain the adapted intervention at scale

### Build sustainable partnerships, capacity, and plans for maintenance

Throughout this guidance we have emphasised involving a range of stakeholders at all stages, with partnerships in place from the start to maximise the likelihood of long term sustainable implementation. Hence, developing capacity, forming plans for implementation and maintenance, and identifying dedicated resources for maintenance will be a focus of these processes. Scalability will be a key consideration from the point of selecting an intervention to adapt. Plans for long term maintenance will ideally be developed incrementally through adaptation, piloting, and evaluation. If the intervention has been implemented in routine practice previously, adaptation teams could seek advice on challenges and solutions from stakeholders who have been involved in implementation and maintenance elsewhere.

### Establish data monitoring systems

Establishing systems to monitor long term implementation and effects will be useful to maintain high quality delivery at scale, and to understand issues such as how reach and uptake increase or fade over time. For example, within the National Exercise Referral Scheme in Wales, routine data analysed 10 years after completion of a randomised trial indicated increased socioeconomic patterning over time in terms of engagement with the programme.^57^ If interventions have been implemented elsewhere, adaptation teams could consider whether harmonisation of monitoring systems is appropriate to enable comparisons across different contexts. As context changes over time, the process of making responsive adaptations will continue after interventions are taken to scale. Building in mechanisms for capturing such adaptations and classifying them as consistent or inconsistent with intervention function,^22^ ^24^ is important to understand if adaptations maintain coherence with intention over time.

## Report the adapted intervention

Unclear reporting of adaptations (which commonly resembles tendencies for limited clarity in reporting the development of new interventions^58^) has inhibited our ability to understand the role of adaptation in reproducing interventions and their effects in new contexts. Several sources of existing guidance are relevant to intervention adaptation, such as TIDIeR-PHP,^29^ which includes recommendations on reporting both planned and unplanned variations in delivery, and the recent FRAME guidance for reporting adaptations.^4^ Several additional reporting items (box 5) for studies involving the adaptation of interventions for new contexts were rated as important in our Delphi surveys or identified in subsequent workshops.

Box 5 **Reporting items for adapted interventions**

- Describe the population health problem being dealt with
- Describe the original intervention and its context and report its evidence base
- Describe the new context, and similarities and differences to the original context
- Describe the rationale, type, and processes undertaken to adapt the intervention, including which stakeholders were involved
- Describe the adapted intervention in detail to enable replication
- Describe how well the adapted intervention was delivered in the new context
- Describe the rationale for the type of re-evaluation
- Describe the role of original intervention developers in the adaptation

## Summary

This new guidance offers a framework and checklist to help researchers, policy and practice stakeholders, funders, and journal editors in undertaking and assessing the adaptation of interventions for a new context, and reporting these transparently. It is a starting point for advancing an ongoing debate, rather than offering a definitive conclusion. Guidance can activate change within knowledge systems, by altering the behaviour of funders, researchers, and other decision makers to produce better, more useful, and efficient, research. In some instances, more research might be needed to understand context before adaptation. However, this guidance might also reduce research waste where, for example, systematic consideration of transferability leads to smaller, more focused evaluations, targeted towards uncertainties. 

We anticipate that our guidance will lead to more systematic and accountable decision making and reporting of adaptation processes, as well as stimulate new thinking and innovation in adaptation research. Over time, an accumulation of adaptation studies based on this guidance and other approaches will help determine what conditions work best in adapting interventions for new contexts, with recommendations refined and firmly grounded in empirical evidence. The guidance was developed through research and engagement with a diverse set of academic, policy, and practice stakeholders. We aimed to achieve broad applicability to interventions in a range of domains, from patient facing interventions within healthcare systems to interventions to improve health in specific settings (eg, schools) or in whole populations. Use of the guidance in specific intervention domains with defined patient or public user groups should involve members of these groups. We welcome feedback on how the guidance might be improved in future updates.

## Notes

Contributors: GM and RE conceived the idea for the ADAPT Study and were coprincipal investigators. Work package 1 (reviews) contributors were ER, AM, LP, AO’C, JS, HL, and BH. Work package 2 (qualitative work) contributors were LC, HL, DC, and PHo. Work package 3 (Delphi) contributors were PC and MC. GM and RE contributed to all work packages, and led work package integration and guidance development, with input from all members. PHa and FK were members of a study advisory group who made authorship level contributions to the work. GM led the drafting of the guidance and this manuscript, with a first draft developed with a core initial writing group including AM, PC, MC, LC, and RE. All authors contributed to the underpinning research and to subsequent drafts of the guidance and manuscript and approved the final version. GM acts as guarantor. The corresponding author attests that all listed authors meet authorship criteria and that no others meeting the criteria have been omitted.

The lead author affirms that the manuscript is an honest, accurate, and transparent account of the study being reported; that no important aspects of the study have been omitted; and that any discrepancies from the study as planned (and, if relevant, registered) have been explained.

Patient and public involvement: This research was done without patient involvement. We involved policy and practice representatives with experience of intervention adaptation in qualitative interviews, the Delphi exercise and our study advisory group.
